# Educated but Unhealthy? Examining Minorities' Diminished Returns

**DOI:** 10.31586/gjeid.2024.1105

**Published:** 2024-11-09

**Authors:** Shervin Assari, Hossein Zare

**Affiliations:** 1 Marginalized-Related Diminished Returns (MDRs) Research Center, Los Angeles, CA, USA; 2 Department of Family Medicine, Charles R. Drew University of Medicine and Science, Los Angeles, CA, USA; 3 Department of Urban Public Health, Charles R. Drew University of Medicine and Science, Los Angeles, CA, USA; 4 Department of Health Policy and Management, Johns Hopkins Bloomberg School of Public Health, Baltimore, MD, USA; 5 School of Business, University of Maryland Global Campus (UMGC), Adelphi, MD, USA

**Keywords:** Self-Rated Health, Educational Attainment, Minorities' Diminished Returns, Racial Disparities, Ethnic Disparities, Latino, Black, Ethnic Groups

## Abstract

**Background:**

Educational attainment is known to improve self-rated health; however, research suggests that these benefits may be less pronounced for racial and ethnic minority groups. The Minorities' Diminished Returns (MDRs) theory posits that the protective effects of resources such as education are weaker for marginalized populations, such as Black and Latino individuals, compared to their White counterparts.

**Objective:**

This study aims to investigate racial and ethnic disparities in the association between years of schooling and self-rated health among U.S. adults, with a focus on understanding the reduced health benefits of education for Black and Latino individuals.

**Methods:**

Using data from the Understanding America Study (UAS; 2014), we conducted a cross-sectional analysis of adults aged 18 and older (N = 6,785). Self-rated health was the outcome, and years of schooling was the primary independent variable. We controlled for sociodemographic factors including age, gender, employment status, immigration status, and marital status. Stratified analyses were conducted by race/ethnicity (Non-Latino White, Non-Latino Black, and Latino). Linear regression models were used to examine the association between years of schooling and self-rated health, and interaction terms were included to assess variation in this relationship across racial/ethnic groups.

**Results:**

While years of schooling was positively associated with better self-rated health overall, the magnitude of this effect was weaker for Black and Latino individuals compared to White individuals. After adjusting for sociodemographic factors, Black and Latino adults reported worse self-rated health for each additional year of schooling, compared to their White counterparts, supporting the MDRs hypothesis.

**Conclusion:**

The findings suggest that while higher educational attainment is protective against worse self-rated health, this protection is not equally distributed across racial and ethnic groups. Black and Latino individuals experience diminished returns from their years of schooling in terms of self-rated health, likely due to structural barriers and social inequalities. Policies addressing health disparities must consider these diminished returns and aim to reduce structural racism and discrimination that undermine the benefits of education for minoritized populations.

## 1. Introduction

Self-rated health (SRH) is a widely used measure in public health research due to its strong predictive validity for morbidity, mortality, and overall well-being [1]. SRH reflects individuals’ subjective assessment of their health, typically measured on a gradient ranging from poor to excellent health [2, 3]. This five-level SRH scale—commonly categorized as *poor*, *fair*, *good*, *very good*, and *excellent*—offers nuanced insight into individuals' health status. Research consistently shows a social gradient in SRH, with higher socioeconomic status (SES), particularly educational attainment, being associated with better SRH outcomes [4, 5].

Educational attainment is a powerful social determinant of health that influences a range of outcomes, including SRH [6]. Individuals with higher levels of education tend to report better health due to several factors: increased health knowledge, improved health behaviors, better employment opportunities, and greater access to healthcare [7-9]. However, recent research highlights that the protective effects of education are not equally experienced across racial and ethnic groups [10-14]. The *Minorities' Diminished Returns (MDRs)* theory [15] posits that the health benefits of education, as well as other SES indicators, are weaker for racial and ethnic minorities compared to White populations.

For example, while higher levels of education are associated with improved SRH for most individuals, Black and Latino individuals often experience diminished returns from their educational attainment [10-16]. Structural racism, discrimination, and unequal access to resources may limit the extent to which these groups can convert their educational advantages into better health outcomes [15]. These diminished returns may manifest in higher stress levels, less secure employment opportunities, and less access to high-quality healthcare, despite equivalent levels of education [15].

This study builds on the MDRs framework [15] by examining the association between years of schooling and SRH across different racial and ethnic groups, focusing on Non-Latino Black, Latino, and Non-Latino White adults. The use of SRH as a five-level gradient provides a more detailed understanding of how individuals perceive their health and how these perceptions vary by educational attainment and race/ethnicity. Specifically, we aim to explore whether the association between education and SRH is weaker for Black and Latino adults compared to White adults, after controlling for key sociodemographic factors such as age, gender, employment status, immigration status, and marital status.

By focusing on racial and ethnic variation in the effects of education on SRH, this study contributes to a growing body of research highlighting the inequitable distribution of health benefits from socioeconomic resources in the United States [10-16]. Understanding these diminished returns is critical for informing public health interventions and policies aimed at reducing health disparities and promoting health equity for all racial and ethnic groups.

## 2. Methods

### 2.1. Design and Setting

The Understanding America Study (UAS) [17-20] is a large, nationally representative, internet-based survey conducted by the University of Southern California (USC). The UAS is designed to gather extensive insights on a wide array of social, economic, and health-related issues across the U.S. population. The study employs probability-based sampling methods, drawing from post-office delivery sequence files to recruit participants. To ensure full inclusivity and representativeness, individuals who do not have internet access are provided with internet-enabled devices, such as tablets, along with internet services, enabling them to participate in the surveys.

### 2.2. Data Collection

The UAS collects detailed data on numerous domains, including well-being, retirement readiness, cognitive functioning, health behaviors, and personality traits. These surveys are administered regularly—either annually or biennially—to capture longitudinal data on participants. In addition to socioeconomic and behavioral variables, UAS includes health-related metrics to provide continuous assessments of the participants' health status over time. The UAS [17-20] provides a rich dataset for examining the relationship between educational attainment and retirement preparedness, particularly when investigating variations by race and ethnicity. Its comprehensive data collection process allows for the exploration of how different demographic factors, such as race/ethnicity, may moderate the association between education and health.

### 2.3. Analytical Sample

Our analytical sample was 6,785, Participants selected from the 2014 UAS wave based on available data on SRH, being Latino or non-Latino, and Black or White. Participants could be immigrant or US born.

### 2.4. Measures

Self-rated health. Self-rated health (SRH) is a commonly used and reliable subjective measure of overall health status, often assessed on a five-point Likert scale ranging from *poor* to *excellent*. This measure has been validated across diverse populations and is predictive of various health outcomes, including morbidity, functional limitations, and mortality [21]. While SRH is typically categorized into discrete levels, treating it as a continuous variable allows for a more nuanced analysis of health gradients, capturing subtle differences in how individuals perceive their health. In this study, we utilized SRH as a continuous measure, ranging from 1 (poor health) to 5 (excellent health), to fully capture the variation in self-reported health across the population and to better quantify the association between years of schooling and SRH across different racial and ethnic groups.

In addition to self-rated health (SRH), the key independent variable in this study is educational attainment, measured as years of schooling. Education is self-reported by participants, capturing the total number of years of formal education completed. This continuous measure allows for precise analysis of the relationship between education and SRH, aligning with previous research that demonstrates education as a powerful predictor of health outcomes. The use of years of schooling as a continuous variable enables us to explore how incremental increases in education influence health perceptions across racial and ethnic groups.

Several covariates were included in the analysis to account for potential confounders. Age was measured in years, and gender was self-reported as male or female. Employment status at the time of the survey was categorized as employed or not employed, providing insight into the participants' current work situation, which is an important determinant of health. Marital status was also self-reported and dichotomized as married or not married, given that marital status has been shown to influence health outcomes through social support and economic stability. Immigration status was self-identified. These variables were included in the models to isolate the independent effect of education on SRH, while accounting for other demographic and socioeconomic factors. Nativity was US-born and non-US non (immigrant).

### 2.5. Data Analysis

To explore racial and ethnic differences in retirement preparedness, initial independent t-tests were conducted to assess variations in retirement preparedness between Latino and non-Latino individuals, as well as between White and Black groups. Additionally, t-tests were used to compare age and educational attainment across these racial and ethnic groups.

Next, linear regression models were employed to examine the relationship between educational attainment (measured by years of schooling) and SRH (a score), while adjusting for key demographic factors such as race, ethnicity, age, gender, employment status, marital status, and nativity (immigration status). Two models were specified for the analysis: Model 1 included educational attainment, race, ethnicity, and /other control variables, without interaction terms. Model 2 incorporated interaction terms between race/ethnicity and years of education to assess whether the relationship between educational attainment and retirement preparedness varied by race and ethnicity.

Results were reported as beta coefficients, along with p-values and 95% confidence intervals (CIs). This analytical approach allowed for a detailed assessment of potential differences in how educational attainment impacts retirement preparedness across racial and ethnic groups, with a particular focus on the Minorities' Diminished Returns (MDRs) theory [22-30]. According to MDRs, the effects of educational attainment on outcomes like retirement preparedness may be less substantial for Latino and Black individuals compared to non-Latino Whites [15, 16, 31-35].

### 2.6. Ethical Considerations

All participants had previously consented to their involvement in UAS-related research as part of their enrollment in the UAS panel. For this specific analysis, the University of Southern California's Institutional Review Board (IRB) required additional informed consent procedures. Participants were explicitly informed that individuals with significant cognitive impairments, who may not fully understand their rights, were excluded from the study. To confirm participants' understanding of their rights, they were asked to complete a brief multiple-choice quiz before giving their final consent. The study received full approval from the USC IRB.

## 3. Results

As shown by Table 1, the sample had a mean age of 48.36 years (SD = 15.67) and an average of 11.10 years of schooling (SD = 2.27). The mean self-rated health (SRH), measured on a 1-5 scale, was 2.56 (SD = 1.0).

Regarding race, 90.6% of the participants were White (n = 6144), and 9.4% were Black (n = 641). In terms of ethnicity, 87.8% were non-Latino (n = 5955), and 12.2% were Latino (n = 830). From all participants, 42.5% were male (n = 2886), and 57.5% were female (n = 3899). Most participants were born in the U.S., with 93.6% being non-immigrants (n = 6350) and 6.4% being immigrants (n = 435). In terms of employment, 58.1% of the sample were working (n = 3944), while 41.8% were not working (n = 2836). Marital status revealed that 57.1% of participants were married (n = 3876), while 42.8% were not married (n = 2906).

As shown by Table 2, years of schooling were significantly associated with lower SRH score (better health) (B = −0.089, 95% CI = −0.100, −0.079, p < 0.001). Being US-born was marginally associated with higher SRH score (worse health) (B = 0.095, 95% CI = −0.002, 0.192, p = 0.055). Employment was significantly associated with lower SRH score (better health) (B = −0.332, 95% CI = −0.382, −0.282, p < 0.001). Being married was significantly associated with lower SRH score (better health) (B = −0.195, 95% CI = −0.242, −0.147, p = 0.000). Latino ethnicity was not significantly associated with SRH (B = 0.043, 95% CI = −0.033, 0.118, p = 0.268). Black ethnicity was not significantly associated with SRH (B = 0.009, 95% CI = −0.070, 0.088, p = 0.827). Older age was significantly associated with higher SRH score (worse health) (B = 0.004, 95% CI = 0.002, 0.005, p < 0.001). Female gender was not significantly associated with SRH (B = 0.009, 95% CI = −0.037, 0.056, p = 0.693).

As shown by Table 3, years of schooling were significantly associated with lower SRH score (B = −0.154, 95% CI = −0.195, −0.113, p < 0.001). The interaction between years of schooling and Black ethnicity was significantly associated with higher SRH score (B = 0.051, 95% CI = 0.015, 0.087, p = 0.006). The interaction between years of schooling and Latino ethnicity was significantly associated with higher SRH score (B = 0.065, 95% CI = 0.037, 0.094, p = 0.000). Employment was significantly associated with lower SRH score (B = −0.336, 95% CI = −0.386, −0.286, p < 0.001). Being married was significantly associated with lower SRH score (B = −0.193, 95% CI = −0.240, −0.145, p < 0.001). Being US-born was not significantly associated with SRH (B = 0.076, 95% CI = −0.021, 0.173, p = 0.127). Older age was significantly associated with higher SRH score (B = 0.004, 95% CI = 0.002, 0.005, p < 0.001). Female gender was not significantly associated with SRH (B = 0.007, 95% CI = −0.040, 0.054, p = 0.771).

## 4. Discussion

This study aimed to examine the racial and ethnic differences in the association between years of schooling and self-rated health (SRH), building on the framework of Minorities' Diminished Returns (MDRs). Specifically, we sought to determine whether the positive association between education and SRH is weaker for Black and Latino individuals compared to their White counterparts. By treating SRH as a continuous variable and controlling for key sociodemographic factors, we were able to provide a nuanced analysis of how educational attainment influences perceived health across these groups.

Our findings indicate that while additional years of schooling are generally associated with better SRH, this protective effect is significantly weaker for Black and Latino individuals compared to White individuals. After controlling for age, gender, employment status, and marital status, Black and Latino participants reported worse health for each additional year of schooling relative to White participants, supporting the MDRs hypothesis. This diminished association suggests that structural inequalities and social barriers may limit the health benefits of education for marginalized racial and ethnic groups.

The literature on SRH consistently shows that it is a valid and reliable measure of overall health [36-46]. Most studies use SRH either as a categorical variable, dividing it into thresholds such as “poor” vs. “good” health, or as a binary outcome. However, treating SRH as a continuous variable, as done in this study, allows for capturing more granular differences in perceived health across individuals. This approach provides a more refined understanding of how factors such as education influence health perceptions across the full spectrum of the SRH scale, from poor to excellent health.

The Minorities’ Diminished Returns (MDRs) theory [14, 23, 28, 29, 32, 47-63] posits that social determinants of health, such as education, income, and employment, yield smaller health benefits for marginalized racial and ethnic groups than for Whites. This study contributes to the growing MDRs literature by providing evidence that the health returns of education are not equally distributed across racial and ethnic groups [64-66]. Prior research has shown that despite attaining higher levels of education, Black and Latino individuals often face barriers that diminish the positive impact of education on various health outcomes [67-72]. Our findings are consistent with this body of work, further demonstrating that the benefits of education on SRH are less pronounced for Black and Latino individuals [73].

Education is typically one of the most robust predictors of health, often associated with better health behaviors, greater health knowledge, and increased access to healthcare [6-9]. In the general population, higher educational attainment is linked to better SRH across the lifespan [6-9]. However, our findings suggest that these benefits do not fully extend to Black and Latino populations. For these groups, years of schooling may not translate into the same level of health improvement, as their access to the social and economic benefits of education may be limited by systemic discrimination, lower-quality education, and fewer opportunities for upward mobility.

The MDRs of education in Black and Latino populations are particularly concerning because education is widely viewed as a critical pathway to reducing health disparities [74-77]. Despite this promise, the weaker health benefits of education for these groups underscore the need to address structural barriers that undermine the protective effects of schooling. Factors such as residential segregation, discrimination in employment and healthcare, and chronic stress associated with racism may explain why Black and Latino individuals experience diminished returns from their education. These systemic issues often prevent them from fully leveraging their educational achievements to improve their health and well-being.

The mechanisms behind MDRs are rooted in structural racism and social inequality [15]. Marginalized groups face a range of stressors, including discrimination, economic instability, and poor access to high-quality education, that collectively limit the positive effects of education on health. Even when Black and Latino individuals achieve higher levels of education, they are often exposed to more adverse working conditions, less stable employment, and higher levels of stress, all of which erode the potential health benefits of education. This ongoing exposure to structural disadvantages likely explains the diminished association between education and SRH observed in this study.

The implications of these findings are significant for public health interventions and policy. Strategies aimed at improving educational attainment may not be sufficient to reduce health disparities if they do not also address the broader structural and systemic barriers that constrain the benefits of education for marginalized groups. Policymakers should focus on creating environments where the social and health returns of education are more equitably distributed. This could involve tackling discrimination in healthcare, improving the quality of education in marginalized communities, and reducing employment inequities that disproportionately affect Black and Latino individuals.

Several limitations should be noted. First, this study is cross-sectional, which limits the ability to draw causal conclusions about the relationship between education and SRH. Longitudinal data would provide stronger evidence of the temporal relationship between educational attainment and health outcomes. Additionally, while we controlled for several key sociodemographic factors, unmeasured confounders such as neighborhood conditions and access to healthcare may have influenced the results. Finally, the study relies on self-reported measures of both education and health, which may be subject to reporting biases.

In conclusion, this study demonstrates that while education is a key determinant of self-rated health, its protective effects are not equally experienced across racial and ethnic groups. Black and Latino individuals face diminished returns from their educational attainment in terms of health, likely due to structural racism and social inequalities. Addressing these disparities requires targeted policies that go beyond improving education alone and instead focus on dismantling the broader systemic barriers that undermine health equity for marginalized populations.

## Figures and Tables

**Table 1.** Descriptive Data

	Mean	SD
Age (Years)	48.36	15.67
Educational Attainment (Years)	11.10	2.27
Self-rated Health (SRH; 1-5)	2.56	1.0
	n	%
**Race**		
White	6144	90.6
Black	641	9.4
**Ethnicity**		
Non-Latino	5955	87.8
Latino	830	12.2
**Gender**		
Male	2886	42.5
Female	3899	57.5
**US Born**		
Immigrant	435	6.4
Non-immigrant	6350	93.6
**Working**		
No	2836	41.8
Yes	3944	58.1
**Married**		
No	2906	42.8
Yes	3876	57.1

Source: Understanding America Study (UAS - 2014)

**Table 2.** Summary of Regression Model without Interaction

Variable	Unstandardized Coefficients (B)	Std. Error	Standardized Coefficients (Beta)	95.0% Confidence Interval (Lower Bound)	95.0% Confidence Interval (Upper Bound)	p (Sig.)
Latino	0.043	0.038	0.014	−0.033	0.118	0.268
Black	0.009	0.040	0.003	−0.070	0.088	0.827
Age	0.004	0.001	0.059	0.002	0.005	< 0.001
Female	0.009	0.024	0.005	−0.037	0.056	0.693
Years of Schooling	−0.089	0.005	−0.203	−0.100	−0.079	< 0.001
US Born	0.095	0.049	0.023	−0.002	0.192	0.055
Employed	−0.332	0.026	−0.164	−0.382	−0.282	< 0.001
Married	−0.195	0.024	−0.097	−0.242	−0.147	< 0.001

Source: Understanding America Study (UAS - 2014) ; Outcome: Self-rated Health (SRH)

**Table 3.** Summary of Regression Model with Interaction

Variable	Unstandardized Coefficients (B)	Std. Error	Standardized Coefficients (Beta)	95.0% Confidence Interval (Lower Bound)	95.0% Confidence Interval (Upper Bound)	p (Sig.)
Latino	−0.647	0.158	−0.212	−0.957	−0.337	< 0.001
Black	−0.542	0.201	−0.159	−0.936	−0.148	0.007
Age	0.004	0.001	0.058	0.002	0.005	< 0.001
Female	0.007	0.024	0.003	−0.040	0.054	0.771
Years of Schooling	−0.154	0.021	−0.351	−0.195	−0.113	< 0.001
US Born	0.076	0.050	0.019	−0.021	0.173	0.127
Employed	−0.336	0.026	−0.166	−0.386	−0.286	< 0.001
Married	−0.193	0.024	−0.096	−0.240	−0.145	< 0.001
Years of Schooling x Black	0.051	0.018	0.200	0.015	0.087	0.006
Years of Schooling x Latino	0.065	0.015	0.228	0.037	0.094	< 0.001

Source: Understanding America Study (UAS - 2014); Outcome: Self-rated Health (SRH)
